# Incidence and outcome of contrast-associated acute kidney injury in a mixed medical-surgical ICU population: a retrospective study

**DOI:** 10.1186/1471-2369-14-31

**Published:** 2013-02-04

**Authors:** Christophe Clec’h, Dominique Razafimandimby, Mehdi Laouisset, Frank Chemouni, Yves Cohen

**Affiliations:** 1 Medical-surgical Intensive Care Unit, Service de réanimation, Hôpital Avicenne, Avicenne teaching Hospital, 125 Route de Stalingrad, Bobigny Cedex 93009, France; 2 INSERM U823, Clinical epidemiology of critically ill patients and airway cancer, Albert Bonniot Institute, Rond-Point de la Chantourne, BP 217, Grenoble 38043, France

**Keywords:** Acute kidney injury, Contrast-associated acute kidney injury, Intensive car unit, Mortality, Outcome

## Abstract

**Background:**

Contrast-enhanced radiographic examinations carry the risk of contrast-associated acute kidney injury (CA-AKI). While CA-AKI is a well-known complication outside the intensive care unit (ICU) setting, data on CA-AKI in ICU patients are scarce. Our aim was to assess the incidence and short-term outcome of CA-AKI in a mixed medical-surgical ICU population.

**Methods:**

We conducted a single-center retrospective analysis between September 2006 and December 2008 on adult patients who underwent a contrast-enhanced computed tomography for urgent diagnostic purposes. CA-AKI was defined as either a relative increment in serum creatinine of ≥ 25% or an absolute increment in serum creatinine of ≥ 0.3 mg/dL within 48 hrs after contrast administration. ICU mortality rates of patients with and without CA-AKI were compared in univariate and multivariate analyses. The need for renal replacement therapy (RRT) was also recorded.

**Results:**

CA-AKI occurred in 24/143 (16.8%) patients. Coexisting risk factors for kidney injury, such as sepsis, nephrotoxic drugs and hemodynamic failure were commonly observed in patients who developed CA-AKI. ICU mortality was significantly higher in patients with than in those without CA-AKI (50% vs 21%, p = 0.004). In multivariate logistic regression, CA-AKI remained associated with ICU mortality (odds ratio: 3.48, 95% confidence interval: 1.10-11.46, p = 0.04). RRT was required in 7 (29.2%) patients with CA-AKI.

**Conclusions:**

In our cohort, CA-AKI was a frequent complication. It was associated with a poor short-term outcome and seemed to occur mainly when multiple risk factors for kidney injury were present. Administration of ICM should be considered as a potential high-risk procedure and not as a routine innocuous practice in ICU patients.

## Background

Acute kidney injury (AKI) is a frequent complication in both general and intensive care unit (ICU) patients, that significantly contributes to morbidity and mortality [1-5].

Among the multiple causes of hospital-acquired AKI, administration of iodinated contrast media (ICM) has been found to be the third commonest one [6], and contrast-associated AKI (CA-AKI) has been reported to be responsible for a 5.5-fold increased risk of death [7].

In the specific ICU setting, administration of ICM to patients with otherwise multiple risk factors for AKI (sepsis, nephrotoxic drugs, and hemodynamic failure) may be even more deleterious. Paradoxically, few studies [8-14] have focused on CA-AKI in these high-risk patients, who very frequently undergo contrast-enhanced computed tomography (CT). Moreover, both the incidence and outcome of CA-AKI vary widely across studies according to case mix and definitions of AKI.

Despite CA-AKI being a well-known complication outside the ICU, the paucity and the discrepancy of data in the ICU justifies additional data. The aim of this study was to evaluate the incidence and short-term outcome of CA-AKI in a mixed medical-surgical ICU population.

## Methods

### Study design and patients

This retrospective study was carried out in the medical-surgical ICU of the Avicenne teaching hospital (Bobigny, France). All adult patients who required a contrast-enhanced CT for urgent diagnostic purposes between September 2006 and December 2008 were eligible for inclusion. Patients with coronary or non coronary angiography were not considered because they do not correspond to our usual patient population. Exclusion criteria were: history of chronic kidney disease, AKI with or without renal replacement therapy (RRT) before administration of ICM, and multiple administrations of ICM. For patients who were admitted more than once to the ICU, only the first ICU stay was included in the analysis.

The decision to perform a contrast-enhanced CT was left at the attending physicians’ discretion. During the whole study period, iso-osmolar media only were used. There were no written protocols for either CA-AKI prevention or dosing of ICM. Indications for and modalities of RRT were also left at the attending physicians’ discretion. No patient received “prophylactic” RRT after administration of ICM.

The study was approved by the local ethics committee (Commission Qualité Sécurité et Ethique des Soins, Avicenne Hospital, Bobigny, France) and conducted in accordance with the declaration of Helsinki. Since the study implied no change in patients’ management and data were anonymously processed, the need for informed consent was waived.

### Definition of CA-AKI

In the absence of consensual definition criteria, CA-AKI was defined as either a relative increment in serum creatinine of ≥ 25%, as in many prior reports [15], or as an absolute increment in serum creatinine of ≥ 0.3 mg/dL (26 μmol/L), as suggested by the Acute Kidney Injury Network [16], within 48 hours after administration of ICM.

The term “contrast-associated” instead of “contrast-induced” AKI was preferred because the development of AKI in the ICU setting is often of multiple origins, thus making hazardous to consider the administration of ICM as the unique cause for AKI. Rather, the nephrotoxicity of ICM may be cumulative (ie, may occur mainly when other risk factors for AKI are present).

### Data collection

For each patient, the following variables were collected:

– Baseline characteristics: age, gender, SAPS II score, Mc Cabe class (class 1, no fatal underlying disease; class 2, underlying disease fatal within 5 years; class 3, underlying disease fatal within 1 year), admission category (medical, scheduled surgery, or unscheduled surgery), and comorbidities (diabetes mellitus, myeloma and other chronic coexisting conditions defined according to the Knaus criteria [17]),

– Serum creatinine values measured on the CT day before administration of ICM, and 24 hours and 48 hours thereafter,

– Additional risk factors for AKI occurring within 48 hours before and after administration of ICM: sepsis, hemodynamic failure and prescription of potential nephrotoxic drugs (aminoglycosides, glycopeptides, trimethoprim/sulfamethoxazole, loop diuretics),

– Presence or absence of preventive measures (*N*-acetylcysteine or isotonic crystalloids), and

– Impact of CA-AKI: need for RRT within 48 hours after administration of ICM, ICU mortality, length of ICU stay and persistent need for renal support on ICU discharge.

### Endpoints

The primary endpoints were the incidence of CA-AKI and short-term outcome of CA-AKI (ie, need for RRT and ICU mortality).

The secondary endpoints were the length of ICU stay, and persistent need for renal support on ICU discharge.

### Statistical analyses

Comparisons between patients with and those without CA-AKI were based on the Fischer’s exact test for categorical variables and on the Wilcoxon’s test for continuous variables.

The effect of CA-AKI on ICU mortality was assessed through a logistic regression model. Specifically, we adjusted for patients’ baseline severity reflected by the SAPS II score and for organ dysfunctions before administration of ICM reflected by the non-renal SOFA score (SOFA - renal component). The goodness of fit and the discrimination of the model were determined by the Hosmer-Lemeshow statistic and the *c* statistic (area under the curve), respectively. Results are shown as adjusted odds ratios with their 95% confidence intervals.

All *p* values were two-tailed, and a *p* value < .05 was considered significant.

Statistical analyses were performed using a statistical software package (SAS, version 9.1; SAS Institute; Cary, NC).

## Results

### Patients

**Figure 1:**
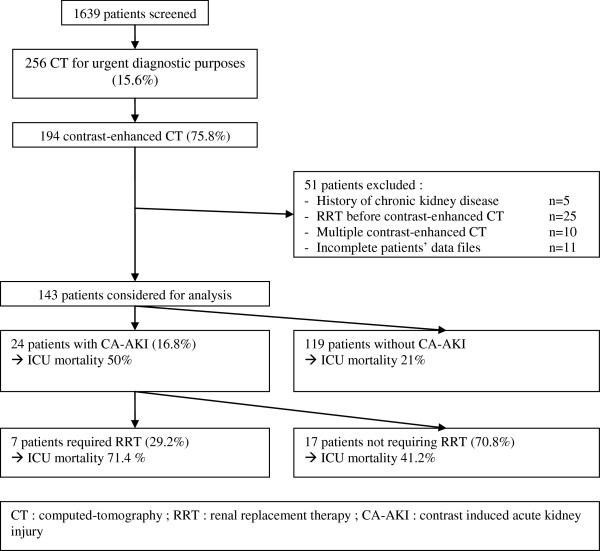
Study flow chart.

Over the study period, 1639 patients were admitted to our unit. Two hundred fifty-six (15.6%) CTs were required for urgent diagnostic purposes, of whom 194 (75.8%) were contrast-enhanced. Forty patients were excluded. In 11 cases, the patient’s data file was incomplete. Finally, 143 patients were considered for analysis (Figure 1).

### Incidence of CA-AKI and comparison of patients with and without CA-AKI

CA-AKI occurred in 24/143 (16.8%) patients (Figure 1).

There was no difference in serum creatinine before CT between patients with and those without CA-AKI but patients with CA-AKI had more risk factors for AKI within 48 hours before administration of ICM, such as sepsis and hemodynamic failure. Preventive measures were rarely used, without any difference between patients with and those without CA-AKI.

Within 48 hours after administration of ICM, the rate of new risk factors for AKI was similar in the two groups (Table 1).

**Table 1:** Differences between patients with and those without contrast-associated acute kidney injury (CA-AKI)

	**No CA-AKI N = 119**	**CA-AKI N = 24**	***P***
Age (yrs)	61 [49–73]	63 [53–78]	0.33
Male sex	74 (62.2)	15 (62.5)	0.98
SAPS II score	40 [28–57]	57 [41–78]	0.001
Admission category			
medical	83 (69.8)	14 (58.3)	0.22
scheduled surgery	11 (9.2)	1 (4.2)	
unscheduled surgery	25 (21)	9 (37.5)	
Mc Cabe class			
1	65 (54.6)	9 (37.5)	0.08
2	38 (31.9)	8 (33.3)	
3	16 (13.5)	7 (29.2)	
Serum creatinine (μmol/L) before administration of ICM	87 [68–120]	90 [64–128]	0.91
Preventive measure	6 (5)	1(4.2)	1
Risk factors for AKI within 48 hrs before ICM administration	64 (53.8)	20 (83.3)	0.01
diabetes mellitus	24 (20.2)	4 (16.7)	1
myeloma	0 (0)	1 (4.2)	0.17
nephrotoxic drugs	22 (18.5)	7 (29.2)	0.26
sepsis	48 (40.3)	16 (66.7)	0.02
hemodynamic failure	14 (11.8)	11 (45.8)	< 0.001
New risk factors for AKI within 48 hrs after ICM administration	36 (30.2)	7 (29.2)	1
nephrotoxic drugs	19 (16)	4 (16.7)	1
sepsis	30 (25.2)	7 (29.2)	0.63
hemodynamic failure	4 (3.4)	1 (4.2)	0.9

Results are expressed as median [interquartile range] or as number (percent).

ICM, iodinated contrast media.

### Association of CA-AKI with renal replacement therapy

RRT was initiated in 7/24 (29.2%) patients with CA-AKI with a median time of 1 day (interquartile range: 0–2) after CA-AKI onset (Figure 1). Patients with CA-AKI who received RRT had similar SAPS II scores and serum creatinine before CT as patients with CA-AKI who did not receive RRT but had more failing organs before CT and were more likely to die in the ICU, although statistical significance was not reached (Table 2). No direct link between administration of ICM and need for RRT could be established due to the absence of consensual indications for RRT and the low number of patients.

**Table 2:** Differences between patients with contrast-associated acute kidney injury who received and who did not receive renal replacement therapy (RRT)

	**No RRT N = 17**	**RRT N = 7**	***P***
SAPS II	58 [44–72]	50 [37–92]	0.97
Non-renal SOFA score before ICM administration	5 [4-6]	7 [5-9]	0.24
Serum creatinine (μmol/L) before ICM administration	90 [63–128]	83 [66–198]	0.80
Risk factors for AKI within 48 hrs before ICM administration	14 (82.3)	6 (85.7)	1
diabetes mellitus	3 (17.7)	1 (14.3)	1
myeloma	1 (5.9)	0 (0)	1
nephrotoxic drugs	5 (29.4)	2 (28.6)	1
sepsis	10 (58.9)	6 (85.7)	0.37
hemodynamic failure	7 (41.2)	4 (57.1)	0.76
New risk factors for AKI within 48 hrs after ICM administration	4 (23.5)	3 (42.9)	0.37
nephrotoxic drugs	3 (17.7)	1 (14.3)	1
sepsis	4 (23.5)	3 (42.9)	0.63
hemodynamic failure	0 (0)	1 (14.3)	0.3
Preventive measure	0 (0)	1 (14.3)	0.3
ICU mortality	7 (41.2)	5 (71.4)	0.37

Results are expressed as median [interquartile range] or as number (percent).

ICM, iodinated contrast media; AKI, acute kidney injury; non-renal SOFA score, SOFA score - renal component.

In the two surviving patients with CA-AKI who received RRT, renal function had recovered on ICU discharge.

### Association of CA-AKI with mortality

Patients with CA-AKI had a significantly higher crude ICU mortality rate than patients without CA-AKI (50% vs 21%, p = 0.004) (Figure 1). After adjustment for patients’ severity, CA-AKI remained an independent risk factor for ICU mortality (Table 3).

**Table 3:** Factors independently associated with intensive care unit mortality

**Variable**	**OR**	**95% CI**	***P***
CA-AKI	3.48	1.10-11.46	0.04
SAPS II score, per point	1.03	1.01-1.05	0.03
Non-renal SOFA score, per point	1.38	1.12-1.71	0.003

Hosmer-Lemeshow chi square = 9.7, p = 0.20, c statistic = 0.80.

CA-AKI, contrast-associated acute kidney injury; OR, odds ratio; CI, confidence interval.

Non-renal SOFA score, SOFA score - renal component.

### Association of CA-AKI with length of ICU stay

Patients with and those without CA-AKI had similar lengths of ICU stay (median in days, [interquartile range]: 8 [4–22] vs 8 [6–23], p = 0.66). Among survivors, patients with CA-AKI had a trend towards increased lengths of ICU stay (median in days, [interquartile range]: 8 [4–18] vs 18 [7–26], p = 0.08).

## Discussion

While CA-AKI has been extensively investigated outside the ICU setting, few studies on CA-AKI in ICU patients have been carried out so far. Moreover, these studies reported discrepant results regarding the incidence and outcome of CA-AKI according to case mix and definitions of AKI [8–14].

In this work, we did not only assess the incidence of CA-AKI but we also assessed the outcome of patients in terms of need for RRT and ICU mortality. We found CA-AKI to be a frequent event in our mixed medical-surgical population. It was associated with a high risk of ICU death, and tended to increase the length of ICU stay. These results are consistent with the ones of Hoste et al [9]. Remarkably, the ICU mortality in CA-AKI patients observed in this study (50%) was about twice the ICU mortality of all-cause AKI reported in recent studies evaluating the prognosis of AKI, as defined by RIFLE criteria [1,3,4]. Another remarkable finding was that most patients with CA-AKI had multiple risk factors for AKI, lending support to the concept of cumulative toxicity (rather than absolute toxicity) of ICM. In addition, RRT failed to reverse renal dysfunction and reduce mortality in CA-AKI patients. Accordingly, administration of ICM should be considered as a potential high-risk procedure and not as a routine innocuous practice in ICU patients, and the technical possibility to put patients on RRT “if necessary” should not be used as an argument to force radiologists to administer ICM.

Although emphasizing the significant burden of CA-AKI in ICU patients, our study has potential limitations. First, we were not able to accurately assess the benefit/risk ratio of contrast-enhanced CT since precise indications of CT and subsequent therapeutic changes were hazardous to extract retrospectively from patients’ files. This important issue deserves further investigation. Second, we did not examine the influence of the volume of ICM and application of preventive measures on the incidence of CA-AKI. The volume of ICM, which has been shown to play a role in the development of CA-AKI, [15] could not be retrospectively retrieved. Regarding preventive measures, they were too rarely used, probably due to the emergency context. It must be stressed, anyhow, that volume expansion and hemodynamic optimization are probably of utmost importance to reduce the risk of CA-AKI, even though the impact of preventive measures in ICU patients needs to be further evaluated. Third, we did not assess the impact of repeated administrations of ICM because of the many confounding factors. Fourth, fluid balance and hemodilution which could have lead to underestimation of CA-AKI incidence (by limiting serum creatinine increase) were not collected. Yet, it must be noticed that this limitation pertains to all studies using the glomerular filtration rate criteria of AKIN or RIFLE definitions. Finally, one may argue that the absence of a “control” group not receiving ICM hinders any definite conclusion as to the actual toxicity of ICM. However, the aim of this study was not to establish the absolute toxicity of ICM but to determine the incidence of CA-AKI and identify potential coexisting risk factors that are important to consider before exposing patients to ICM. Moreover, it must be stressed that patients are undoubtedly their best controls, and that random variations in serum creatinine among “control” patients, that would equate to AKI [18], may expose to biased estimation of the true risk associated with ICM and erroneous inference. In addition, the chronological order of events, the time constraint of 48 hours, and the similar rate of new risk factors for renal dysfunction in patients with and without CA-AKI suggest a cause-effect relationship between administration of ICM and AKI.

## Conclusions

In our mixed medical-surgical ICU population requiring contrast-enhanced CTs for urgent diagnostic purposes, CA-AKI was a frequent and severe complication. In keeping with the few available retrospective data, our study indicates that the administration of ICM in patients with otherwise multiple risk factors for AKI should be considered with caution, all the more as CA-AKI may not only impair short-term but also long-term prognosis [9,19].

Large prospective trials on ICU patients are warranted to more accurately assess the benefit/risk ratio of contrast-enhanced CTs, the role of preventive measures, and the impact of repeated administrations of ICM.

## Abbreviations

AKI: Acute kidney injury; ICU: Intensive care unit; CA-AKI: Contrast-associated acute kidney injury; ICM: Iodinated contrast media; CT: Computed tomography.

## Competing interests

The authors declare that they have no competing interests.

## Authors’ contribution

CC designed the study and wrote the manuscript; CC performed the statistical analyses; DR, ML, FC and YC participated in the collection of data and critically revised the manuscript for important intellectual content. All authors read and approved the final manuscript.

## Pre-publication history

The pre-publication history for this paper can be accessed here:

http://www.biomedcentral.com/1471-2369/14/31/prepub
